# Phage PPPL-1, A New Biological Agent to Control Bacterial Canker Caused by *Pseudomonas syringae* pv. *actinidiae* in Kiwifruit

**DOI:** 10.3390/antibiotics10050554

**Published:** 2021-05-10

**Authors:** Yu-Rim Song, Nguyen Trung Vu, Jungkum Park, In Sun Hwang, Hyeon-Ju Jeong, Youn-Sup Cho, Chang-Sik Oh

**Affiliations:** 1 Department of Horticultural Biotechnology, College of Life Science, Kyung Hee University, Yongin 17104, Korea; yulimy@khu.ac.kr (Y.-R.S.); nguyen12sh@gmail.com (N.T.V.); jungkuum@naver.com (J.P.); hongkong10@hanmail.net (I.S.H.); 2 Fruit Research Institute, Jeollanamdo Agricultural Research and Extension Services, Haenam-gun 59021, Korea; pob1256@korea.kr (H.-J.J.); aktis@korea.kr (Y.-S.C.); 3 Graduate School of Biotechnology, Kyung Hee University, Yongin 17104, Korea

**Keywords:** bacterial canker, disease control, kiwifruit, phage, *Pseudomonas syringae* pv. *actinidiae*

## Abstract

*Pseudomonas syringae* pv. *actinidiae* (Psa) is a Gram-negative bacterium that causes bacterial canker disease in kiwifruit. Copper or antibiotics have been used in orchards to control this disease, but the recent emergence of antibiotic-resistant Psa has called for the development of a new control agent. We previously reported that the bacteriophage (or phage) PPPL-1 showed antibacterial activity for both biovar 2 and 3 of Psa. To investigate the possibility of PPPL-1 to control bacterial canker in kiwifruit, we further tested the efficacy of PPPL-1 and its phage cocktail with two other phages on suppressing disease development under greenhouse conditions using 6 weeks old kiwifruit plants. Our results showed that the disease control efficacy of PPPL-1 treatment was statistically similar to those of phage cocktail treatment or Agrimycin^TM^, which contains streptomycin and oxytetracycline antibiotics as active ingredients. Moreover, PPPL-1 could successfully kill streptomycin-resistant Psa isolates, of which the treatment of Buramycin^TM^ carrying only streptomycin as an active ingredient had no effect in vitro. The phage PPPL-1 was further characterized, and stability assays showed that the phage was stable in the field soil and at low temperature of 0 ± 2 °C. In addition, the phage could be scaled up quickly up to 10^10^ pfu/mL at 12 h later from initial multiplicity of infection of 0.000005. Our results indicate that PPPL-1 phage is a useful candidate as a biocontrol agent and could be a tool to control the bacterial canker in kiwifruit by Psa infection in the field conditions.

## 1. Introduction

Bacterial canker caused by Psa has been considered as the most devastating disease in both *Actinidia deliciosa* (green kiwifruit) and *Actinidia chinensis* (yellow kiwifruit) [1,2,3]. The disease symptoms can occur on the various organs of kiwifruit plants such as red ooze on cane and trunk, dark brown spots with the yellowish halos on leaves, wilting vines, and necrosis in flowers [4,5]. Among them, the wide death of vines leads to the most economic loss [6]. The serious damage by Psa infection on the kiwifruit industry was reported in the major kiwifruit growing countries such as China, Italy, and New Zealand [7,8,9,10].

Psa was first isolated in Japan in 1984 [11], and sporadic outbreaks were reported in Korea [4,12], Portugal [13], Spain [14], France [15], Turkey [16], Slovenia [17], Greece [18], and Georgia [19]. Based on geographical, genetic, and biological characteristics, Psa strains can be grouped into biovars 1, 2, 3, 5, and 6 [20]. Biovar 4 was reclassified as *P. syringae* pathovar *actinidifoliorum* [21]. In Korea, several Psa strains belonging to biovar 2 were isolated from green kiwifruit cv. ‘Hayward’ (e.g., JYS5) and yellow kiwifruit cv. ‘Hort16A’ (e.g., KBE9) [4]. Biovar 3 strains, which was first isolated in Italy in 2008 [1], also appeared in Korea (e.g., SYS1) [22]. Recently, biovar 3 strains have been found in Europe, New Zealand, Chile, and China, resulting in severe damage in the international kiwifruit industry [23,24,25].

Currently, only a few effective therapeutic definitive methods are demonstrated for canker disease in kiwifruit. Copper or streptomycin products have been traditionally used [26], but Psa strains resistant to copper and streptomycin have been reported in several countries including Korea [27,28,29]. Moreover, continuous usage or environmental contamination with these chemicals significantly contributes to generation of resistant bacteria. Hence, alternatives methods to manage bacterial canker in kiwifruit are needed.

A phage is a virus that infects and kills host bacterial cells, and it is mostly species specific. It is not harmful to human and environment, thus it has been considered as an eco-friendly agent to control plant pathogenic bacteria, in particular, antibiotic-resistant pathogenic bacteria [30,31]. Phages have the self-replicating property and high host specificity. These characteristics make them promising as an alternative method to antibiotics for control of bacterial canker [32]. To control phytopathogenic bacteria, several phage-based products such as AgriPhage™ (Salt Lake City, UT, USA) have already been developed and commercialized, and many studies showing significant biocontrol efficacy of phages for the management of the several important phytopathogenic bacteria in recent years have been reported [33].

Although the large number of phages targeting to Psa were isolated and characterized [34,35,36,37], only a few of them such as *Pseudomonas* phage φ6 were demonstrated to show their control efficacy ex vivo on bacterial canker [38]. We previously reported virulent phages, PPPL-1, KHUφ34, and KHUφ38, which could kill Psa strains belonging to both biovar 2 and 3 and other *P. syringae* pathovars [37,39]. PPPL-1 and KHUφ38 belong to *Podoviridae* family, while KHUφ34 is a member of *Myoviridae* family. Additionally, PPPL-1 genome analysis illustrated that it is a virulent phage with genes encoding a class II holin and Rz-like lysis protein, but no genes relating to lysogenic cycle [39]. Furthermore, this phage was stable under diverse environmental conditions where kiwifruit trees are growing. Thus, we aimed to further examine the potential of PPPL-1 phage in controlling bacterial canker disease in kiwifruit as well as in managing antibiotic-resistant Psa isolates. Furthermore, the combination of PPPL-1 with KHUφ34 and KHUφ38 in controlling bacterial canker disease in kiwifruit was also evaluated.

## 2. Results

### 2.1. Control Efficacy of PPPL-1 Phage to Prevent Bacterial Canker in Planta

**Figure 1:**
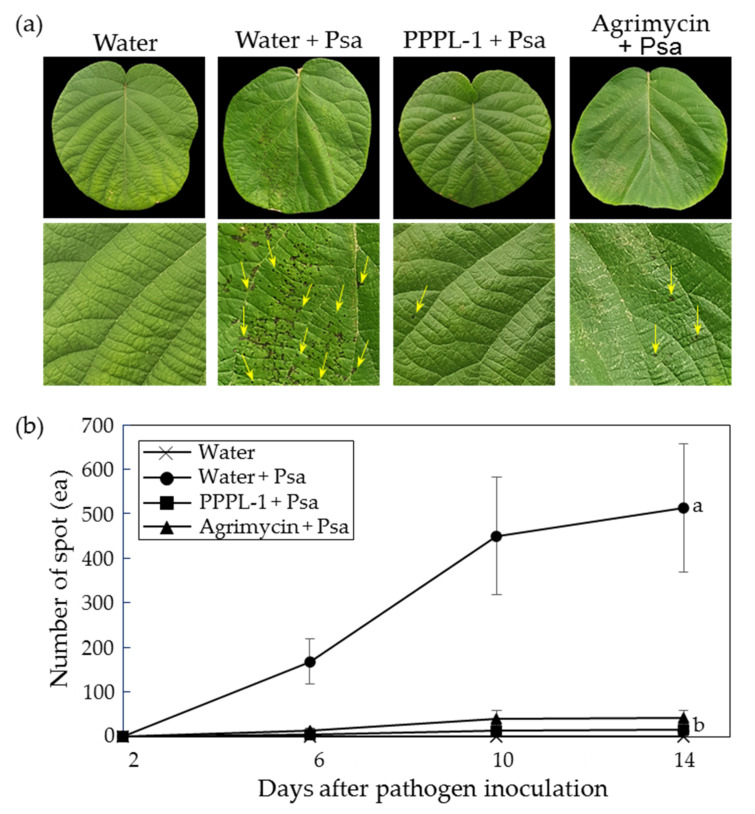
Control efficacy of PPPL-1 phage treatment against bacterial canker caused by *Pseudomonas syringae* pv. *actinidiae* (Psa) KBE9 and SYS1 mixture in planta. (**a**) The kiwifruit leaves 10 days after pathogen inoculation (dai) with phages or antibiotic pesticide. Bottom figures are enlarged photographs of top leaves. The yellow arrows indicate symptomatic spots. The sterilized water was used for dilution of phages and bacteria. Water, PPPL-1 (10^8^ pfu/mL), or Agrimycin™ (0.4 g/L) was applied 2 h before infection with Psa (10^8^ cfu/mL). (**b**) The mean of numbers of symptomatic spots on the treated leaves (*n* = 12). The error bars indicate the standard error, and the different alphabets in the next of lines indicate the different groups based on significant differences at *p* < 0.05 by Duncan’s multiple range test at 14 dai.

To test the control efficacy of PPPL-1 phage against bacterial canker in kiwifruit, 10^8^ pfu/mL of phages (equivalent to MOI 1.0) were first treated on both sides of 12 kiwifruit leaves of 5 plants under greenhouse conditions, followed by application of Psa KBE9 (biovar 2) and SYS1 (biovar 3) mixture 2 h after phage treatment. The PPPL-1 phage application significantly protected the treated leaves with Psa, based on the reduction in visible symptomatic spots compared to the untreated one (Figure 1a). Indeed, the number of visible symptomatic spots in the PPPL-1-pretreated leaves maintained at a lower rate around 16.7 ± 4.63 (mean ± standard error) spots after 14 days after inoculation (dai), while it continuously increased and reached to an average of 512.9 ± 144.35 spots at 14 dai in the untreated leaves (Figure 1b). Furthermore, the disease incidence in the PPPL-1-pretreated leaves was not statistically different from that in leaves treated with commercial antibiotic product-Agrimycin™ (approximately 41.7 spots ± 16.83) (Figure 1b). These assays were repeated three times with the similar results. These results indicate that the pretreatment of PPPL-1 phage can efficiently control bacterial canker in kiwifruit as much as the treatment of the antibiotics product.

**Figure 2:**
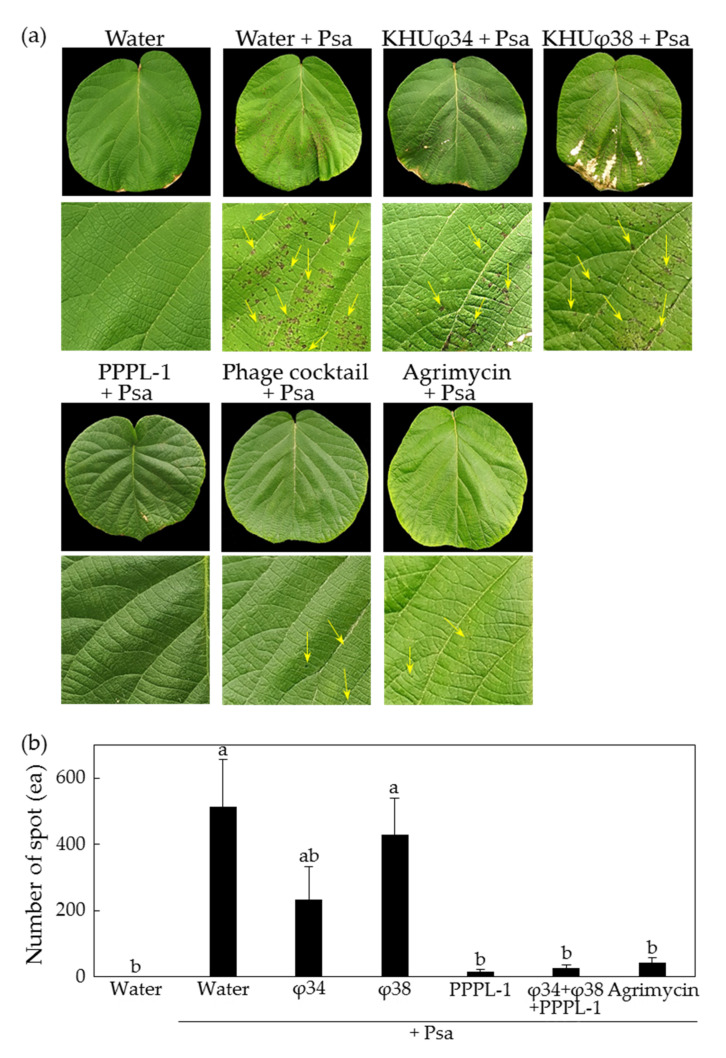
Control efficacy of PPPL-1 phage compared to KHUφ34, KHUφ38, or phage cocktail with all three phages against bacterial canker caused by *Pseudomonas syringae* pv. *actinidiae* (Psa) KBE9 and SYS1 mixture in planta. (**a**) The kiwifruit leaves 10 days after Psa inoculation (dai) with phages or antibiotic pesticide. Bottom figures of each treatment are enlarged photographs of top leaves. The yellow arrows indicate symptomatic spots. The sterilized water was used for dilution of phages and bacteria. Buffer, each phage (10^8^ pfu/mL), or Agrimycin™ (0.4 g/L) was treated 2 h before treatment with bacterial suspension (10^8^ cfu/mL). Mock and buffer are sterilized water. (**b**) The mean of number of symptomatic spots on the treated leaves (*n* = 12). The error bars indicate the standard error, and the different letters on top of each bar indicate the different groups based on significant differences at *p* < 0.05 by Duncan’s multiple range test at 10 dai.

Previously, we reported other Psa phages, KHUφ34 and KHUφ38, which belong to Myoviridae and Podoviridae, respectively, and their lytic activity against Psa SYS1 (biovar 3) was less than that against Psa KBE9 (biovar 2) in vitro [37]. Therefore, we tried to compare their control efficacy against Psa KBE9 and SYS1 mixture in planta. Consistently to the previous result, in the presence of Psa KBE9 infection, the individual treatment of KHUφ34 and KHUφ38 phages showed less effects compared to PPPL-1 in planta, but the phage cocktail with three phages showed the similar efficacy to PPPL-1 phage alone (Figure 2). While the leaves treated with Psa only showed an average of 512.9 ± 144.35 symptomatic spots at 14 dai, the leaves pretreated with KHUφ34 and KHUφ38 showed 234.17 ± 98.46 and 428.33 ± 112.15 spots, compared with only 16.67 ± 4.63 and 27.08 ± 10.16 spots by pretreatment with PPPL-1 and the phage cocktail, respectively (Figure 2b). These assays were repeated twice with the similar results. Overall, these results indicate that the control efficacy of PPPL-1 phage is significantly similar to phage cocktail treatment and better than those of two other phages, consistent with in vitro lytic activity.

### 2.2. Concentration and Treatment Timing of PPPL-1 Phage for Efficient Control of Bacterial Canker in Planta

**Figure 3:**
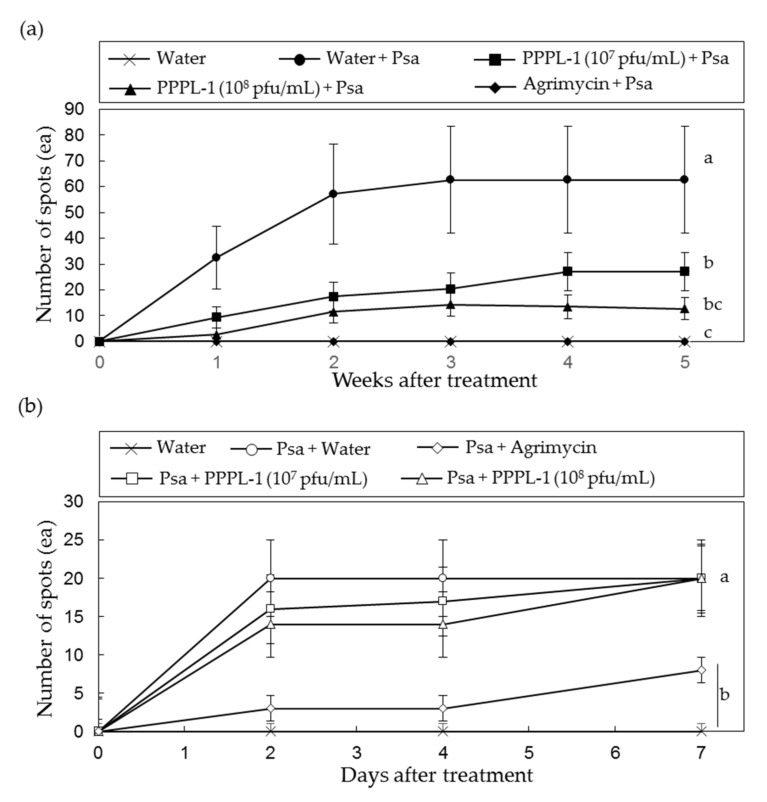
Effects of phage concentration and timing of phage treatment on control efficacy of PPPL-1 phage treatment against bacterial canker caused by *Pseudomonas syringae* pv. *actinidiae* (Psa) KBE9 in kiwifruit leaves. (**a**) Leaves were treated with phages at 10^7^ and 10^8^ pfu/mL by brushing on both sides of leaves, then Psa was applied on the same sides 2 h later. (**b**) Leaves were inoculated with Psa on both sides of leaves by brushing, then phages at 10^7^ and 10^8^ pfu/mL were applied on the same sides by brushing. The front treatment of each treatment label was first applied, and the back one was treated 2 h later. The error bars indicate the standard error (*n* = 20), and the different alphabets in the next of lines indicate the different groups based on significant differences at *p* < 0.05 by Duncan’s multiple range test at 5 weeks or 7 days after treatment.

To examine if phage concentration could be reduced for disease control, the control efficacy of MOI 0.1 was compared with that of MOI 1.0 in 20 leaves of 5 plants under greenhouse conditions. While leaves treated with Psa KBE9 only showed 62.7 ± 20.7 spots 5 weeks after treatment, about 12.6 ± 4.29 and 27.1 ± 7.29 spots from MOI 1.0 and MOI 0.1, respectively, were observed (Figure 3a). These assays were repeated twice with the similar results. These results indicate that, although both MOI treatments significantly reduced the disease severity by Psa in kiwifruit leaves, MOI 1.0 was more efficient, and it was statistically similar to Agrimycin^TM^ treatment.

In addition to the prophylactic efficacy of PPPL-1 phage, its therapeutic efficacy with MOI 1.0 was also examined. The plants were inoculated with Psa bacteria and then phages were applied 2 h later. As a result, the control efficacy of phage treatment on kiwifruit leaves after pathogen treatment exhibited statistically no difference from that of no phage treatment (Figure 3b). These results indicate no therapeutic efficacy of PPPL-1 phage for application in planta.

### 2.3. Antibacterial Effects of PPPL-1 Phage on Streptomycin-Resistant Psa Isolates In Vitro

**Figure 4:**
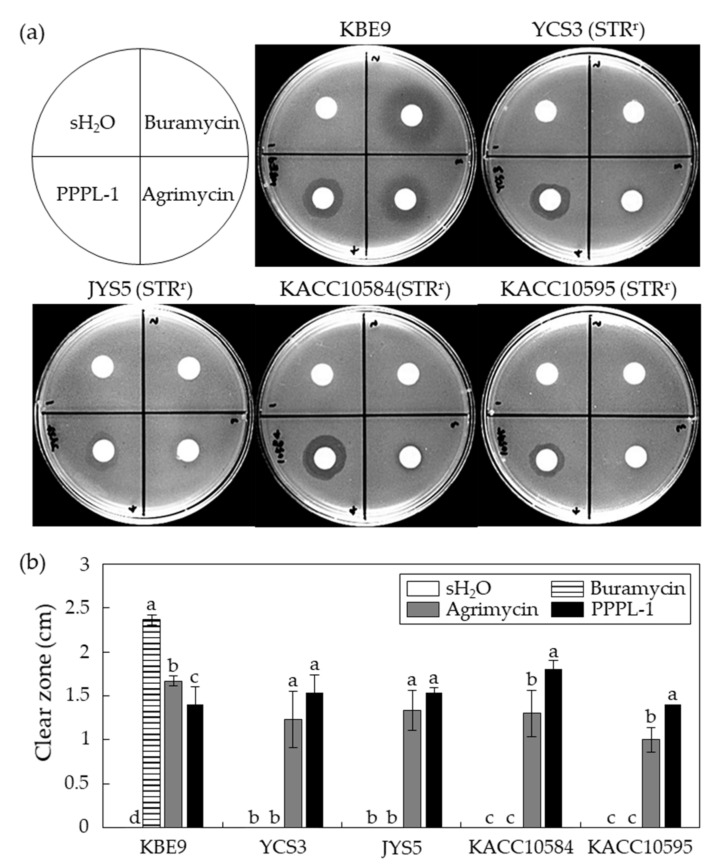
Antibacterial effects of PPPL-1 on streptomycin-resistant Psa isolates. (**a**) The images of plates showing clear zones. The most top left figure showed the treatment of other figures. (**b**) Length of clear zone caused by each treatment. The error bars indicate the standard deviation (*n* = 3), and the alphabets on top of each bar indicate the different groups based on significant differences at *p* < 0.05 by Duncan’s multiple range test in each strain. sH_2_O, sterilized water; Buramycin™ (1.25 g/L); Agrimycin™ (0.4 g/L); PPPL-1 (10^8^ pfu/mL).

Streptomycin-based products are mainly used for control of Psa [26,28]. However, many studies have reported the emergence of streptomycin-resistant Psa isolates [27,29,40]. Four streptomycin-resistant Psa strains isolated from South Korea (YCS3, JYS5, KACC10584, and KACC10595) were used to examine the antibacterial effects of PPPL-1 against them in vitro. For this assay, Buramycin^TM^ containing a streptomycin as an active compound and Agrimycin™ containing both streptomycin and oxytetracycline as active compounds were used as controls. Buramycin^TM^ did not suppress bacterial growth of streptomycin-resistant Psa isolates at all, while it caused the formation of clear zone against only Psa strain KBE9, a streptomycin-sensitive isolate (Figure 4a,b). In contrast, both Agrimycin™ and PPPL-1 phage formed clear zones against all Psa strains, and their antibacterial activities were statistically very similar at *p* < 0.05, although the sizes of clear zones slightly varied among Psa isolates. These results indicate that PPPL-1 phage could be used to control streptomycin-resistant Psa isolates like antibiotics products.

### 2.4. Stability of PPPL-1 Phage in the Field Soil and Low Temperature

**Figure 5:**
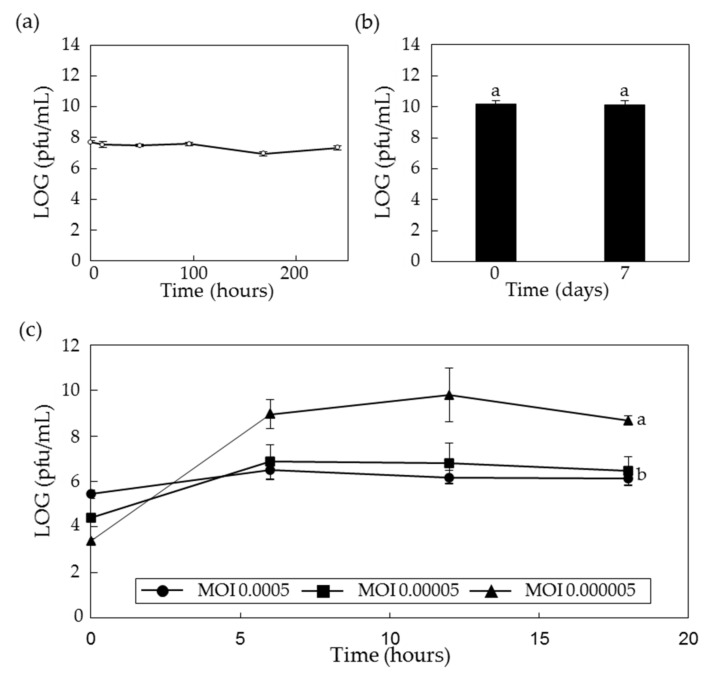
Stability of PPPL-1 phage in the field soil at 26 °C (**a**) and low temperature (0–2 °C) (**b**) and also minimum MOI for scaled-up production (**c**). The phages of 10^8^ pfu/mL for (**a**) and 10^10^ pfu/mL for (**b**) were used for assays. The phage was incubated with host bacteria (OD_600_ = 0.5) at three different MOIs, and the phage concentration was measured at the indicated time points (**c**). Error bars indicate standard deviations of three replicates (*n* = 3), and the alphabets on the top of error bars (**a**) or in the next lines (**b**) indicate the different groups based on significant differences at *p* < 0.05 by Duncan’s multiple range test.

In the previous report, we showed that the lytic activity of PPPL-1 phage is stable under the field temperature (average temperature in kiwifruit-growing regions in Korea over the year: 0 to 26 °C) and pH 4–11 [39]. However, here, we checked how long the lytic activity of PPPL-1 phage can be sustained in the field soil at 26 °C. First, to check the viability of PPPL-1 phage inside the field soil, 10^8^ pfu/mL of PPPL-1 phage was inoculated in the field soil collected from the kiwifruit orchard in Korea. The PPPL-1 phage kept its lytic activity against Psa for more than 240 h in the field soil at 26 °C (Figure 5a). For long-term storage, whether the lytic activity of PPPL-1 phage can be kept stable at low-temperature (0 ± 2 °C) or not is critical. Therefore, about 2 × 10^10^ pfu/mL of PPPL-1 phage solution as the initial titer was kept at this temperature. The data shows that PPPL-1 phage was very stable for more than 7 days at low temperature (Figure 5b). These results indicate that PPPL-1 phage can be used in the field where kiwifruit trees are growing and can be kept at low temperature for storage.

### 2.5. Optimal Conditions for Scaled-Up Production of PPPL-1 Phage

The optimal conditions for scaled-up production of PPPL-1 phage need to be determined, and we tested different parameters to optimize phage titers. Two of critical conditions for efficient massive production of the phage are the optimal stage or concentration of host bacteria and the minimum MOI. First, we checked the optimal bacterial stage for efficient phage production with MOI 0.1. The data highlighted that 0.5 of OD_600_ (approximately 5 × 10^8^ cfu/mL) was more efficient than 0.2 or 1.0 of OD_600_ (data now shown). Next, we checked the minimum MOI with 0.5 of OD_600_ of host bacteria. For this, we set up three different MOIs, MOI 0.0005, 0.00005, and 0.000005, and checked phage titers at several time points. The phage titer in all three MOIs increased during the first 6 h after inoculation of PPPL-1 phage (Figure 5c). However, only the phage titer in MOI 0.000005 increased more until 12 h after inoculation, and it reached to the highest amount (Figure 5c). These results indicate that MOI 0.000005 with OD_600_ 0.5 of host bacteria in liquid medium is optimal for scaling up production of PPPL-1 phage.

## 3. Discussion

Bacterial canker caused by Psa is a destructive disease of kiwifruit and further causes serious economic loss of kiwifruit production worldwide [41]. Recently, a series of papers about the practical bacterial disease control with Pseudomonas phage have been published. However, these studies focus on demonstrating phage activity in vitro, while a few studies to date have previously investigated phage activity both in vitro and ex vivo [38,42]. One of the challenges in biocontrol with phages is the incompatibility of phage efficacy under in vitro and in vivo conditions [33]. However, in this study, we demonstrate the efficacy of PPPL-1 phage in suppressing canker disease in vivo.

The results of PPPL-1 application in kiwifruit plants before Psa infection under greenhouse conditions demonstrated disease suppression as effective as Agrimycin™—a commercial antibiotic-based product (Figure 1 and Figure 2). Thus, this phage might be a promising biological agent for control of bacterial canker disease. However, when the phage was applied after Psa infection, it was unable to suppress disease development (Figure 3b). Unlike our results, the application of a phage cocktail with four phages, CHF1, CHF7, CHF9, and CHF21, in 2-year-old kiwifruit plants (cv. ‘Hayward’) 1 h post infection with Psa significantly suppressed both bacterial growth and disease development after 30 days [42]. The MOI used in this study was 10, which was 10- or 100-fold more than those in our study. Moreover, Psa titer was 100-fold less than that of our study. The usage of higher concentration of phages and less titer of Psa in the in vivo experiment might result in significant control efficacy. Therefore, we suggest applying this phage early spring before the disease is occurred and should not be used after pathogen infects kiwifruit trees in the field orchards. The timing to apply chemical products for control of bacterial canker in field is very critical [43].

The application of phages in field faces with different challenges such as tolerance to environmental conditions [33]. PPPL-1 phage is stable by the way it is treated or under the environment in kiwifruit cultivation regions [39]. This previous study showed that PPPL-1 phage was demonstrated to be stable up to 40 °C, at pH range of 3–11, and under UV-A. Furthermore, in this study, we showed its longevity in the orchard soil at 26 °C and its stability at low temperature (Figure 5). Our results together with the previous studies support the potential of PPPL-1 phage for bacterial canker control in field where kiwifruit trees are growing. The stability of phages at natural environment in crop-growing regions is one of critical factors for good control efficacy against bacterial diseases. There are many cases where phages are stable in the crop-growing conditions in field [42,44,45].

Currently, phage cocktail has emerged as a solution to overcome the limitation of single phage treatment [33]. Wang et al. [46] explained three different ways that the phage cocktail decreased the bacterial wilt incidence in tomato including (1) individually infecting and killing target bacteria, thus reducing the pathogen density, (2) the slow development of pathogen strains via enforcing phage-resistant development, or (3) encouraging the development of antagonistic bacterial species. Furthermore, to use phage cocktail may slow down the appearance of resistant pathogens if phages in phage cocktail are genetically and morphologically different. Our phage cocktail, KHUφ34, KHUφ38, and PPPL-1, could suppress disease by the mixed Psa infection and showed no statistical difference with PPPL-1 treatment alone in suppressing disease (Figure 3). Therefore, it was failed to see the advantage of phage cocktail for disease control in this study, probably because the treatment with a single phage, PPPL-1, was so efficient. However, at least, there was no negative effects of three phage cocktail in kiwifruit leaves. Because biovar 2 and 3 of Psa are present in Korea and also there is possibility of appearance of resistant pathogen variants against PPPL-1 phage, the phage cocktail mixture should be considered for long-term treatment.

Traditionally, antibiotics have been used to control pathogenic bacteria in not only agriculture but also food processing and human therapy. However, the risk of appearance of antibiotic-resistant pathogens was warned. In the case of bacterial canker disease, streptomycin-based pesticides have been used to control Psa, and streptomycin-resistant strains were also reported [29]. PPPL-1 phage successfully inhibited the growth of several streptomycin-resistant Psa strains isolated in South Korea, compared to two streptomycin-based pesticides-Buramycin™ or Agrimycin™ (Figure 3). Thus, PPPL-1 phage might be used for controlling streptomycin-resistant Psa strains. Furthermore, the combination of PPPL-1 with antibiotics-based pesticides might also enhance the control efficacy of the current antibiotic application only and also reduce the usage of antibiotics-based pesticides. However, further experiments are needed to confirm these possibilities.

Although so many papers demonstrated the efficacy of phages in plant disease control, there are only a few commercial phage-based products such as AgriPhage^TM^, Erwiphage, and Biolyses available worldwide [33]. To determine the best condition for processing of phage products, the concentration of host bacteria and the minimum MOI should be considered and selected. In this study, 0.5 of OD_600_ of host bacteria and MOI of 0.000005 were shown to be the suitable condition for scaled-up production. The relative high cost of phage application compared to the conventional methods is generally originated from the scaled-up production step, and this leads to the difficulty in phage therapy in field conditions [47]. However, the rapid increase in PPPL-1 concentration up to approximately 10^10^ pfu/mL within 12 h might be suitable for scaled-up production within short-term period, thus contributing to the price reduction. These features of PPPL-1 phage will be very useful to make a phage product for commercialization.

## 4. Materials and Methods

### 4.1. Growth Conditions of Bacterial Strains

Six Psa strains including KBE9, SYS1, KACC10584, and KACC10595, isolated from *A. chinensis* cultivar (cv.); ‘Hort16A’ in South Korea; and JYS5 and YCS3 isolated from *A. deliciosa* cv. ‘Hayward’ in South Korea [4] were used in this study. All strains corresponded to biovar 2 except SYS1 (biovar 3). For experimental purpose, a single colony of each strain on Tryptic Soy broth Agar (TSA; Difco, Franklin Lakes, NJ, USA) plate was used for culturing it in 5 mL of liquid Tryptic Soy Broth (TSB; Difco, Franklin Lakes, NJ, USA) in a shaking incubator at 140 rpm and 26 °C for 18 h. The streptomycin-resistant strains (JYS5, YCS3, KACC10584, and KACC10595; [29]) were cultured on media with 50 µg/mL of streptomycin (Duchefa Biochemie, RV Haarlem, The Netherlands).

### 4.2. Phage Lysate Preparation

The phages PPPL-1, KHUφ34, and KHUφ38 [37,39] were stored in sodium chloride-magnesium sulfate (SM) buffer (50 mM Tris-HCl, 100 mM NaCl, and 10 mM MgSO_4_·7H_2_O) at 4 °C for routine use and at −80 °C by glycerol stock for long-term storage. A single plaque of each phage was collected by recovery from glycerol stock using the previously described methods of plaque assays [48] and then resuspended in SM buffer. Briefly, the phage stock solution was inoculated to melted 5 mL TSA containing 0.4% agar (~42 °C) and 100 µL of the bacterial suspension and then poured on TSA solid plate and incubated at 26 °C overnight after properly solidifying.

For phage lysate propagation, 100 µL of plaque resuspension (~10^8^ pfu/mL) was inoculated with 5 mL of overnight (18 h) liquid culture of Psa KBE9 (OD_600_ = 0.5–0.6) in a shaking incubator at 140 rpm for 6 h. Then, the lysate was collected by centrifugation for 5 min at 8000× *g* to remove the remained bacteria as well as its debris. If necessary, the supernatant was treated by 1% chloroform for 30 min, and then the chloroform was removed by centrifugation for 15 min at 3000× *g*. Finally, the supernatant was filtered with 0.22 μm pore size of PVDF syringe filter (Futecs, Deajeon, Korea) and stored in 4 °C. To determine the phage lysate concentration, its 10-fold diluted series was dotted on the soft TSA (0.4% agar) plate inoculated with 100 μL of Psa KBE9 (OD_600_ = 0.5–0.6). Its concentration was calculated by counting the number of plaques formed in the dotted area.

### 4.3. Scaled-Up Production and Precipitation of Phages

To determine the optimal stage of host bacteria for scaled production, the overnight culture of Psa strain KBE9 was diluted 1000-fold in 400 mL liquid medium, and they were grown up to 0.2 (~2 × 10^8^ cfu/mL), 0.5 (~5 × 10^8^ cfu/mL), and 1.0 (~10^9^ cfu/mL) of optical density at 600 nm (OD_600_). Then, the PPPL-1 phage for multiplicity of infection (MOI) 0.1 was added to the bacterial suspension, and its titer was checked 12 h later. Next, to determine the minimum MOI for scaled-up production, 400 mL bacterial suspension of Psa strain KBE9 (OD_600_ = 0.5) was inoculated with 400 µL of 2.5 × 10^6^, 2.5 × 10^7^, and 2.5 × 10^8^ pfu/mL of the phage lysate to reach MOI 0.0005, 0.00005, and 0.000005. After 0, 6, 12, and 18 h of shaking incubation at 26 °C, the phage lysate was collected by centrifugation at 8000 rpm for 10 min and filtration using filter system (0.22 μm; Corning, NY, USA) with vacuum pump system, and the phage titer was calculated by the plaque assay method.

To get higher phage titer, the phage lysate was precipitated using 10% polyethylene glycol 6000 (Daejung Chemical & metals, Siheung, Korea) supplemented with 1 M NaCl (LPS solution, Daejeon, Korea) as final concentrations. After overnight incubation at 4 °C, the pellets were collected by centrifugation at 4 °C and 9000× *g* for 20 min and then resuspended with 2 mL of SM buffer. Finally, 0.1 M KCl was added to the suspension for well separation of phages from the pellets, and the suspension was centrifuged at 12,000× *g* and 4 °C for 10 min.

### 4.4. Control Efficacy Test of PPPL-1 Phage In Vivo

The grafted kiwifruit plants with *A. chinensis* cv. ‘Haehyang’ (originated from the highly susceptible cv. ‘Hongyang’ [3]) as scion and *A. deliciosa* cv. ‘Hayward’ as rootstock were planted in 6 L pots in the greenhouse (15–25 °C) for 6 weeks. For evaluating the control efficacy of PPPL-1 phage in vivo, 12 leaves (about 15 cm in diameter) of 5 plants were treated with phage resuspension (10^8^ pfu/mL) on both sides using the silicon brusher. After 2 h, the bacteria suspension (OD_600_ = 0.1, ~10^8^ cfu/mL) of Psa KBE9 and SYS1 mixture in sterilized tap water (10 mM MgCl_2_ buffer was not used because it caused necrosis in kiwifruit leaves) was treated using the same method. For positive control, leaves were treated with Agrimycin^TM^ (0.4 g/L, SUNGBO Chemicals, Seoul, Korea), and sterilized tap water was used as mock treatment. Moreover, the control efficacy of PPPL-1 phage was compared to the individual KHUφ34 and KHUφ38 phages [38] and also to phage cocktail with all three phages. For phage cocktail, the same amount of each phage was mixed to reach the final concentration of each phage to 10^8^ pfu/mL. To enhance the attachment of treated phage and bacterial cells, 0.02% Silwet L-77 (Momentive, NY, USA) as final concentration was added to all treatments. The treated leaves were observed for 2 weeks, and the number of leaf spots were counted to evaluate the control efficacy.

To optimize the amount of PPPL-1 phage for treatment, two phage concentrations (10^7^ and 10^8^ pfu/mL) were examined in 20 leaves of 5 plants at 2 h before or after inoculation of Psa KBE9. The experiments were performed, as described above. The treated plants were observed for 5 weeks or 7 days.

### 4.5. Antibacterial Effect of PPPL-1 Phage by Filter Paper Disc Method In Vitro

The antibacterial activity of PPPL-1 phage was tested for comparison with recommended pesticides against streptomycin-resistant Psa using disc diffusion test. Briefly, the sterilized filter paper discs (Ø8 mm; Advantech, Taipei, Taiwan) were placed on TSA plates incubated with 5 mL of melting soft agar and 100 µL of either Psa KBE9 suspension (OD_600_ = 0.5) or streptomycin-resistant Psa strains such as YCS3, JYS5, KACC10584, and KACC10595. After completely solidifying, 40 µL of Buramycin™ (1.25 g/L; Farm Hannong, Seoul, Korea), Agrimycin™ (0.4 g/L) or PPPL-1 (10^8^ pfu/mL) was dropped onto each disc. After complete drying, each plate was sealed and incubated at 26 °C. The diameter (cm) of clear zones was measured as the indication of antibacterial activity.

### 4.6. Stability Test of PPPL-1 Phage in the Field Soil and Low Temperature

For stability test in soil, 10^8^ pfu/g of PPPL-1 phage was inoculated to the field soil collected from kiwifruit orchard and then incubated at 26 °C. The live phage titer was determined at 0, 1, 2, 4, and 7 days after inoculation using plaque assay against Psa strain KBE9. For stability at low temperature, 1 mL phage suspension (~10^10^ pfu/mL) was kept at 0–2 °C, and the live phage titer was measured at 7 days after incubation.

### 4.7. Statistical Analysis

To statistically analyze all results, Duncan’s multiple range test (*p* < 0.05) was performed with SAS (version 9.4 for Windows; SAS Institute, Cary, NC, USA). All experiments were repeated more than twice using three or more plants in each assay.

## Data Availability

All data are contained within the article.
